# Biomimetic Keratin-Coated Gold Nanoparticles for Photo-Thermal Therapy in a 3D Bioprinted Glioblastoma Tumor Model

**DOI:** 10.3390/ijms23179528

**Published:** 2022-08-23

**Authors:** Maila Chirivì, Claudia Bearzi, Paolo Rosa, Selenia Miglietta, Francesca Petronella, Elena De Falco, Antonella Calogero, Roberto Pani, Vincenzo Petrozza, Giovanni Perotto, Roberto Rizzi, Luciano De Sio

**Affiliations:** 1 Department of Molecular Medicine, Sapienza University, Viale Regina Elena, 324, 00161 Rome, Italy; 2 UOC Neurology, Fondazione Ca’Granda, Ospedale Maggiore Policlinico, Via F. Sforza, 28, 20122 Milan, Italy; 3 Institute for Biomedical Technologies, National Research Council (ITB-CNR), Segrate, 20090 Milan, Italy; 4 Fondazione Istituto Nazionale di Genetica Molecolare, 20122 Milan, Italy; 5 Department of Medico-Surgical Sciences and Biotechnologies, Research Center for Biophotonics, Sapienza University of Rome, Corso della Repubblica 79, 04100 Latina, Italy; 6 Department of Anatomy, Histology, Forensic Medicine and Orthopaedics, Sapienza University of Rome, 00185 Rome, Italy; 7 Institute of Crystallography, National Research Council, (CNR-IC), Via Salaria Km 29, 300, Monterotondo, 00015 Rome, Italy; 8 Mediterranea Cardiocentro, 80122 Napoli, Italy; 9 Smart Materials, Istituto Italiano di Tecnologia, Via Morego 30, 16163 Genoa, Italy

**Keywords:** photo-thermal therapy, 3D bioprinting, plasmonics, cancer, optics

## Abstract

Before entering human clinical studies to evaluate their safety and effectiveness, new drugs and novel medical treatments are subject to extensive animal testing that are expensive and time-consuming. By contrast, advanced technologies enable the development of animal-free models that allow the efficacy of innovative therapies to be studied without sacrificing animals, while providing helpful information and details. We report on the powerful combination of 3D bioprinting (3DB) and photo-thermal therapy (PTT) applications. To this end, we realize a 3DB construct consisting of glioblastoma U87-MG cells in a 3D geometry, incorporating biomimetic keratin-coated gold nanoparticles (Ker-AuNPs) as a photo-thermal agent. The resulting plasmonic 3DB structures exhibit a homogeneous cell distribution throughout the entire volume while promoting the localization of Ker-AuNPs within the cells. A 3D immunofluorescence assay and transmission electron microscopy (TEM) confirm the uniform distribution of fluorescent-labeled Ker-AuNPs in the volume and their capability to enter the cells. Laser-assisted (λ = 532 nm) PTT experiments demonstrate the extraordinary ability of Ker-AuNPs to generate heating, producing the highest temperature rise of about 16 °C in less than 2 min.

## 1. Introduction

Photo-thermal therapy (PTT) is a minimally invasive cancer treatment realized by combining photo-responsive nanomaterials and suitable light sources. PTT relies on the capability of photosensitizing agents to generate highly localized thermal heating for the selective thermal ablation of tumors [1]. Several nanomaterials-based photosensitizers have been used over the past years, such as organic dyes [2], carbon dots, graphene, and plasmonic nanoparticles (NPs) [3]. Ideally, a photosensitizing agent should be designed to possess a large absorption cross-section, low intrinsic toxicity, be soluble in physiological liquids, and have easy surface functionalization, to provide biocompatibility and to target specific cells. From a functional standpoint, plasmonic nanomaterials such as Au [4] and Ag NPs [5] are exceptional materials because they possess the intrinsic property of localized plasmonic resonance (LPR), an oscillation that occurs due to the interaction of a visible/near-infrared light source and the electrons localized at the metallic/dielectric interface. Associated with the LPR effect, a substantial temperature increase conveys the extraordinary photo-thermal properties of the plasmonic NPs that have been largely exploited for PTT-based applications [4]. Biocompatible Au NPs are essential for PTT applications, thanks to their stability, biocompatibility, and high photo-thermal efficiency. However, the capping agent used to stabilize the Au NPs might induce intrinsic toxicity [6,7]. Several biopolymers such as bovine serum albumin (BSA) [8] and human serum albumin (HSA) [9] have been employed to minimize such risks, enhancing both stability and biocompatibility. In this regard, we have reported a new generation of Au NPs coated with keratin (Ker-AuNPs) [10,11]. Ker-AuNPs show high biocompatibility and excellent photo-thermal properties. We have demonstrated [11] that in a 2D culture, Ker-AuNPs do not affect the cell viability of human U87-MG glioblastoma cells up to 72 h. In addition, keratin provides stability in biological environments, and more importantly, is an excellent platform to promote functionalization with specific biomolecules or receptors (e.g., antibodies) for selective targeting.

In the past few years, PTT combined with multi-modality treatments such as chemotherapy [12], immunotherapy [13], and photodynamic therapy [14] have received much attention for realizing synergistic therapies that combine localized treatment, which help to reduce the primary tumors while controlling circulating cancer cells. However, to test the synergistic effect of PTT-assisted multitherapy according to the heterogeneity and biological complexity of primary tumors, integrated 3D in vitro models are necessary, as the 2D cultures do not reproduce the complex microenvironment and mechanical features of cancer faithfully [15]. More importantly, results obtained by combining 2D cancer models and NPs have been revealed to be not very useful for in vivo applications, because of the complexity of the tumor microenvironment [16,17,18]. 

Three-dimensional bioprinting (3DB) is a validated and valuable technology that utilizes bioinks mixed with living cells for 3D printing in a layer-by-layer fashion, tissue, complex tumor morphologies, and organs, using a bottoms-up approach [19,20]. In 3D bioprinted constructs, cells are organized onto a rigid substrate mimicking the biological architecture desired and the interactions among different cell populations.

Taking a step forward, after testing the biological and functional features of Ker-AuNPs in a 2D glioblastoma multiforme model [11], in this manuscript, we developed an advanced model by combining the 3DP technique with biomimetic Ker-AuNPs to perform 3DB-PTT experiments. We have characterized plasmonic 3DB constructs using morphological, optical, and thermo-optical analysis and viability assays, revealing high biocompatibility and excellent photo-thermal properties for Ker-AuNPs embedded in the 3DB glioblastoma U87-MG construct.

## 2. Results

**Figure 1:**
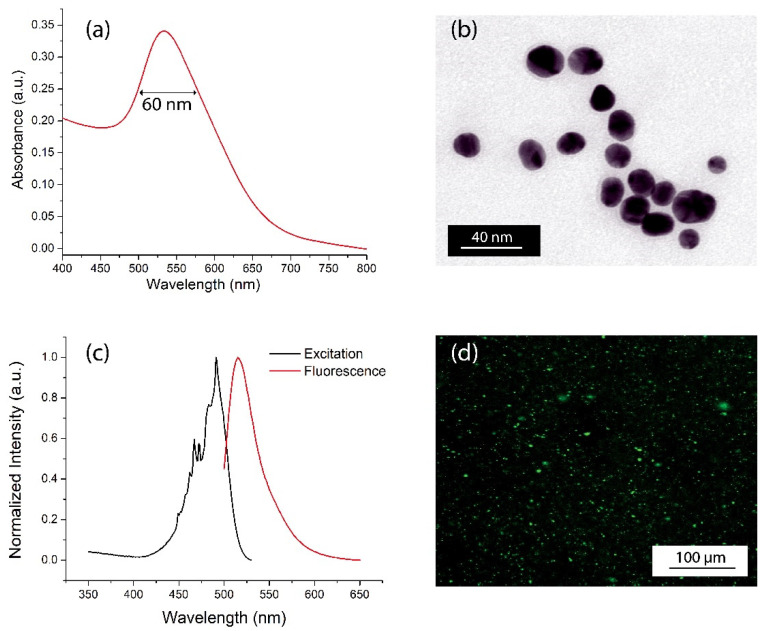
Spectroscopic and morphological characterization. Spectral response (**a**) and representative TEM image (**b**) of Ker-AuNPs. Emission spectrum (**c**) and fluorescence microscope image (**d**) of fluorescent-labeled Ker-AuNPs.

The absorption spectrum of the Ker-AuNPs (Figure 1a) shows an absorption band centered at 534 nm, due to the LPR effect associated with AuNPs, with a full width half maximum (FWHM) of about 60 nm (Figure 1a). The spectral characterization confirms that Ker-AuNPs are spherical, with a very low polydispersity and an average diameter of about 25 nm, as demonstrated by the TEM analysis in Figure 1b. 

To enable the Ker-AuNPs’ identification in the 3DB construct, Ker-AuNPs were labeled with the FITC fluorophore.

The emission spectrum of fluorescent-labeled Ker-AuNPs (Figure 1c, red curve) highlights a sharp emission at 542 nm, thus confirming successful conjugation between the FITC fluorophores and the keratin layer surrounding the AuNPs. The fluorescence of the Ker-AuNPs was also visually proven via fluorescence microscopy characterization. Indeed, Figure 1d shows well dispersed green spots associated with the drop-casted Ker-AuNPs.

**Figure 2:**
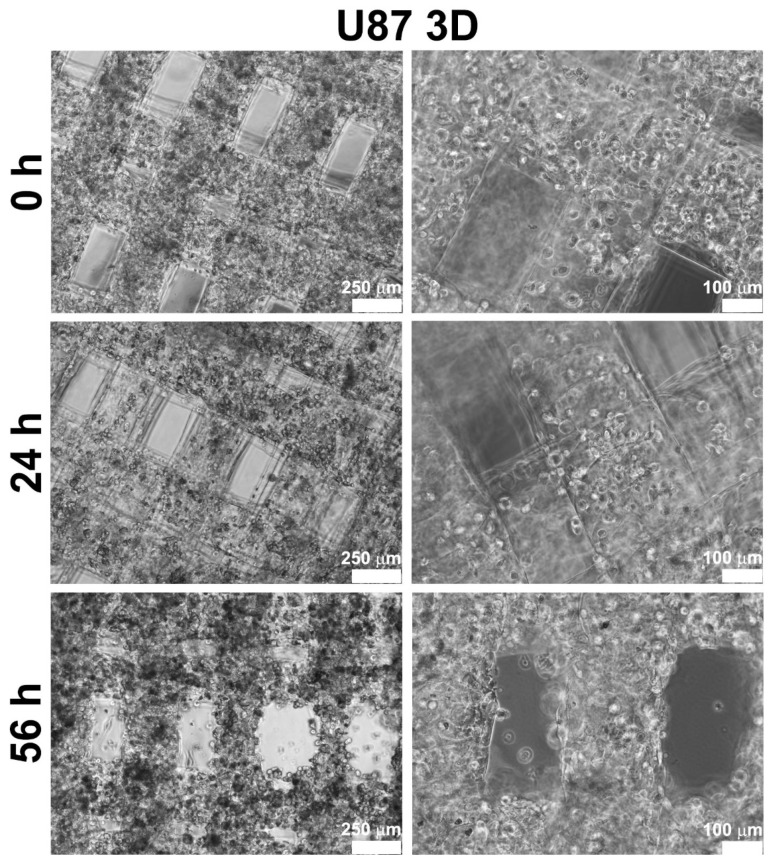
Bright-field images of 3DB constructs. Representative micrographs of U87-MG cells grown in 3DB constructs after 0, 24, and 56 h of culture. Scale bars represent 250 μm and 100 μm.

Glioblastoma U87-MG cells were bioprinted alone or in combination with Ker-AuNPs. After the bioprinting process, U87-MG cells appeared (with and without Ker-AuNPs) as single cells homogeneously distributed throughout the constructs (Figure 2, upper panel). During the time course (at 24 h and 56 h), cells proliferated, colonizing the structure created by the GelMA bioink, as confirmed in Figure 2 (central and lower panel). Notably, after 56 h of culture, the cells maximized their proliferation and aligned along the bioprinted fibers. 

**Figure 3:**
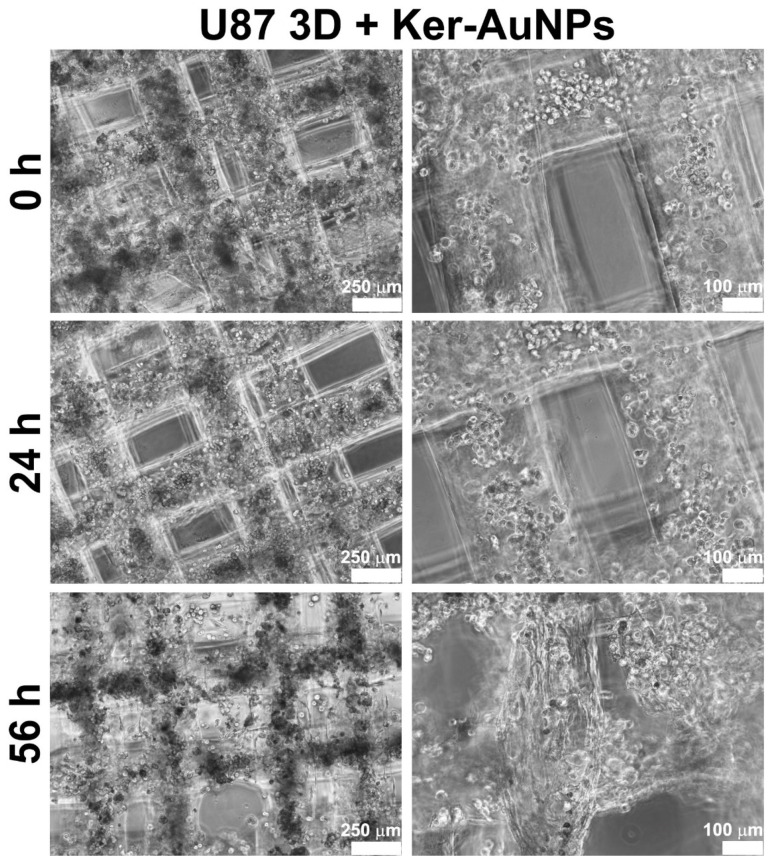
Bright-field images of 3DB constructs. Representative micrographs of U87-MG cells grown in 3DB constructs after 0, 24, and 56 h of culture in presence of Ker-AuNPs. Scale bars represent 250 μm and 100 μm.

Moreover, cell proliferation occurred especially in the presence of Ker-AuNPs, as shown in the lower panel of Figure 3. These results suggest that the Ker-AuNPs did not alter their cell proliferation and spreading within the supporting biomaterial.

**Figure 4:**
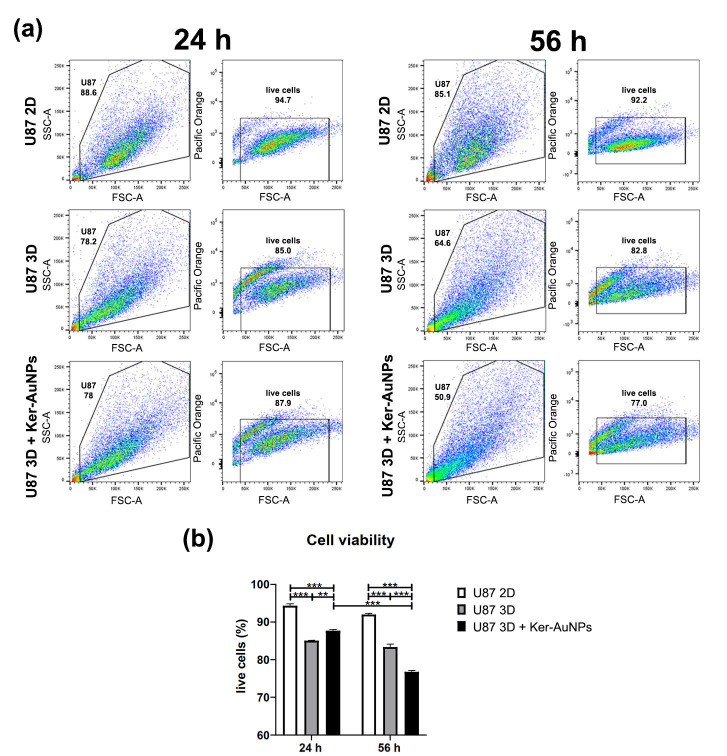
Cell viability. FACS analysis (**a**) of U87-MG cultured in the 2D standard condition and in 3DB constructs, with or without Ker-AuNPs, after 24 (**left** panel) and 56 (**right** panel) h. The quadrant plots show cells identified by physical parameters using side scatter (SSC-A) and forward scatter (FSC-A). Pacific orange marker was used to detect live cells. Percentage of live cells (**b**) in the different culture conditions after 24 and 56 h. ** *p* < 0.002, *** *p* < 0.001 were considered statistically significant. n = 3 for each experimental group.

To strengthen this observation, the 3DB constructs were digested, and cultures were isolated to assess the cell viability via FACS analysis (Figure 4a). The results were compared with the U87-MG 2D culture, and therefore, in the absence of the bioprinting process. The transition from 2D to 3D culture reduced the cell viability (Figure 4b) to less than 10% after 24 h; also, in the 3D cultures, the presence of Ker-AuNPs did not further affect viability (2D = 94.35%; 3D = 85%; U87 3D + Ker-AuNPs = 87.7%). However, a moderate difference was observed only after 56 h of culture for the viability of U87-MG co-cultured with Ker-AuNPs (*p* < 0.001) (Figure 4b). Specifically, the percentage of cell viability in combination with Ker-AuNPs decreased by 8.5 and 15.25 percent, compared to the 3D and 2D conditions, respectively (2D = 92%; 3D = 83.35%; U87 + Ker-AuNPs = 76.75%). 

**Figure 5:**
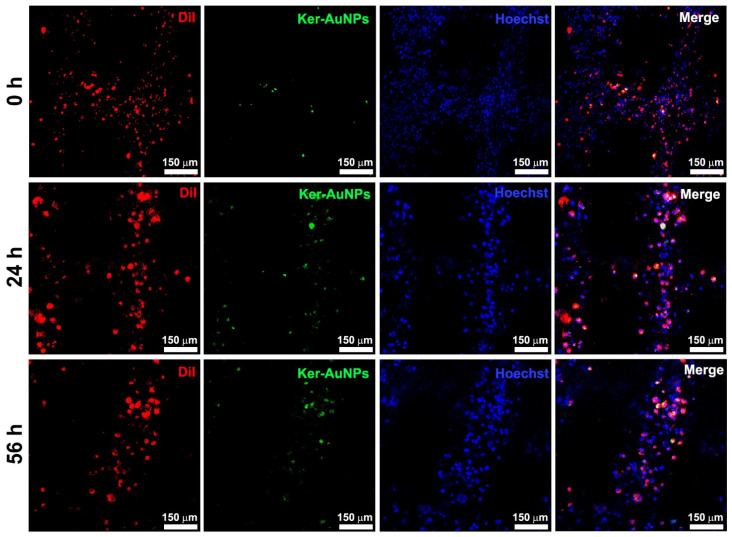
Immunofluorescence assay. Representative images of 3DB construct containing U87, in combination with FITC-labeled Ker-AuNPs after 0, 24, and 56 h of culture. The cells were stained Supplementary Video with DiI (red), while the Ker-AuNPs were detected in green. Nuclei were stained with Hoechst (blue). Scale bars represent 150 μm.

Immunofluorescence analysis after 0, 24, and 56 h of culture was performed to evaluate the localization of Ker-AuNPs within the bioprinted constructs. The representative images (Figure 5) show that the biomaterial provided crucial support to prevent the Ker-AuNPs’ leakage from the constructs. Moreover, the Ker-AuNPs were more localized within the cells after 56 h of culture (Figure 5). As we demonstrated [11], the Ker-AuNPs’ cellular uptake rate and kinetics rate yields a sigmoidal-shaped curve that reaches a plateau after 50 h of incubation. Therefore, it is reasonable to assess that 3DP and a longer incubation time promote the localization of Ker-AuNPs within the cells.

**Figure 6:**
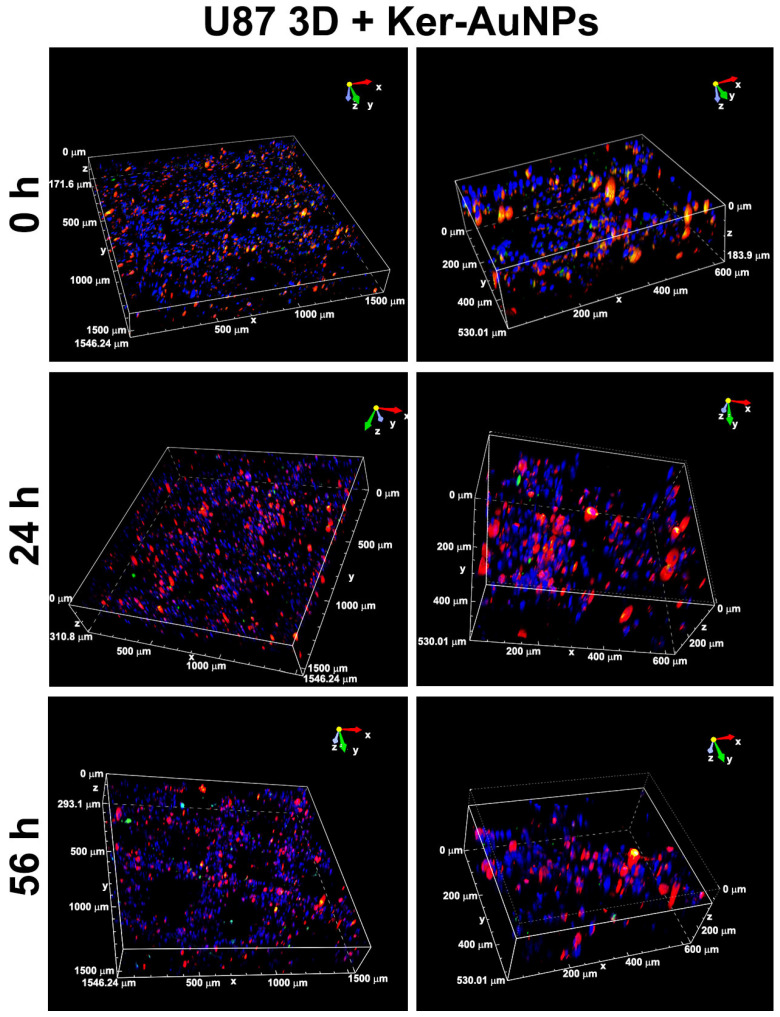
Volumetric 3D reconstruction. Presented are 3D rendering images (**left** panel) and the relative magnification (**right** panel) of bioprinted constructs containing U87 stained with DiI (red) and FITC-labeled Ker-AuNPs. Nuclei were counterstained with Hoechst.

The immunofluorescence analysis (single image acquisition) reported in Figure 5 did not allow an in-depth visualization of the Ker-AuNPs’ distribution in the volume. Thus, a 3D volumetric reconstruction (300 μm^3^) and zooming through Nis-Element software were performed. The 3D rendering images (Figure 6) confirmed the accumulation of Ker-AuNPs in cell clusters, especially after 56 h of culture, coherent with the results observed in Figure 5. 

**Figure 7:**
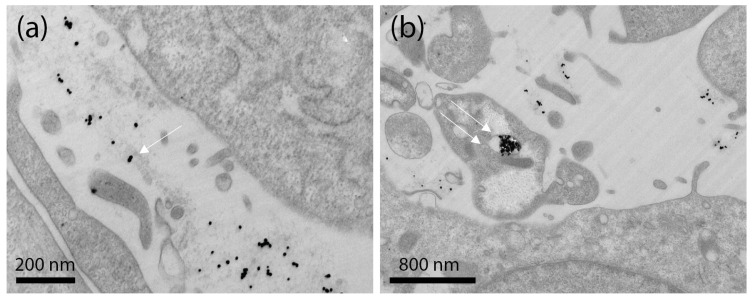
Ultrastructural TEM analysis of Ker-AuNPs in U87-MG cells. Ker-AuNPs were found both outside the cell’s membrane (**a**) and inside small vesicles (**b**).

Ultrastructural TEM analysis of Ker-AuNPs in U87-MG cells showed that Ker-AuNPs made contact with the plasma membrane (Figure 7a, single arrow), occurring in plasma membrane invaginations, and entered the cell enclosed in endosomes. Therefore, the Ker-AuNPs internalized via endocytosis/phagocytosis were trapped in vacuolar structures (Figure 7b, double arrow). The surface of U87-MG was characterized by small protrusions of the plasmalemma. Some cells showed large outgrowths protruding in the external space, usually near cell lateral borders. The formation of these structures is associated with the ability of U87-MG cells to undergo endocytosis/phagocytosis. Notably, the internalization of the Ker-AuNPs did not affect cell morphology, as the nucleus, mitochondria, Golgi apparatus, and rough endoplasmic reticulum were intact and undamaged.

**Figure 8:**
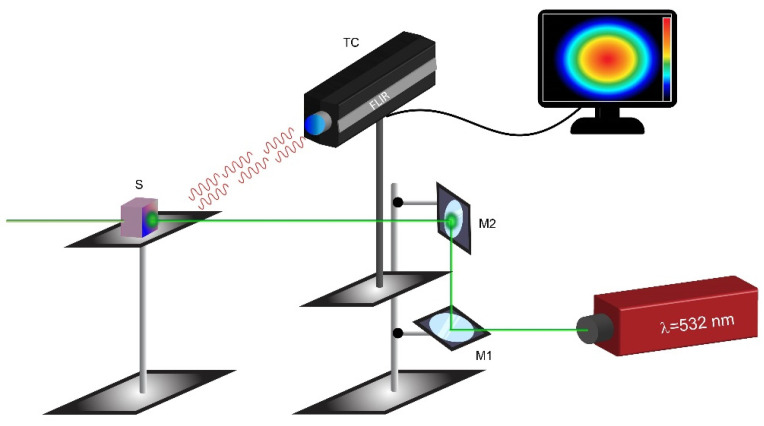
Schematic of the experimental optical setup used for investigating the photo-thermal properties of the 3DB structures. The laser beam impinges on the 3DB structure (S), producing photo-thermal heating, measured using a thermal camera (TC). M1 and M2 are two broadband metallic mirrors.

Laser-assisted PTT experiments were performed to verify the ability of Ker-AuNPs to produce photothermal heating in the 3DB structures. The photothermal investigations were performed using the optical setup depicted in Figure 8.

**Figure 9:**
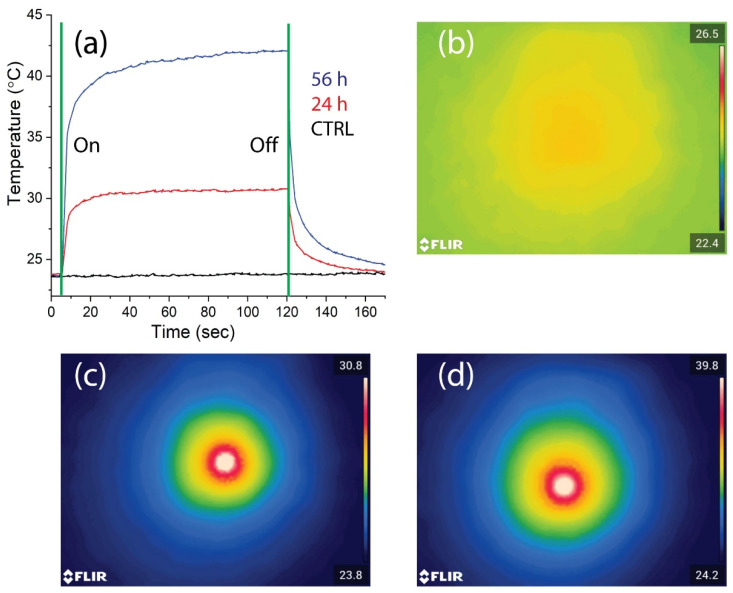
Photo-thermal experiments. Time–temperature dependence (**a**) of the 3DB constructs without (black curve) and with Ker-AuNPs incubated for 24 h (red curve) and 56 h (blue curve), along with the corresponding thermal images (**b**), CTRL; (**c**), 24 h; ((**d**), 56 h) acquired after 120 s of laser illumination (I = 9.1 W/cm^2^).

As shown in Figure 9a, depicting the time–temperature dependence of the samples upon laser beam illumination, the 3DB structures cultured with Ker-AuNPs for 24 and 56 h increased in temperature by about 6 °C (Figure 9a, red curve) and 16 °C (Figure 9a, blue curve), respectively, compared to the control sample (3DB structure culture for 56 h without Ker-AuNPs), which exhibited a negligible effect (Figure 9a, black curve). The results were confirmed by the corresponding thermal images reported in the same Figure 9b–d. Interestingly, the 3DB sample cultured for 56 h was able to double in temperature, and increase >2 times higher than the sample cultured at 24 h.

## 3. Discussion

To date, one of the main issues with the employment of NPs is their toxicity when employed in in vivo models, as they generate toxicity for normal living cells in the tumor microenvironment. Usually, 2D models represent the previous step for testing the delivery and efficacy of NPs. However, they cannot simulate the tissue architecture. More importantly, 2D models lack prediction, as most chemotherapeutic agents are highly effective in culture but not under physiological conditions. This aspect represents a gap to fill, to support the employment of NPs in cancer applications. In this regard, 3D models have displayed lower toxicity and are significantly correlated with in vivo animal studies.

In this study, Ker-AuNPs’ new biomimetic and stimuli-responsive nanomaterials have been combined with 3DB technology. Ker-AuNPs, besides possessing excellent morphological, optical, and thermo-optical properties, exhibit very high biocompatibility, as we have already demonstrated [11]. Ker-AuNPs were able to sustain the cell viability of U87-MG cells for up to 56 h in the 3D model, coherent with the full integration of AuNPs in cell clusters within the construct maximized at the same time point, using a potential endocytosis-based mechanism (Ker-AuNPs’ lack of specific targeting molecules), as shown via 3D volumetric resolution and TEM analysis, respectively. These results confirm that the Ker-AuNPs did not hamper the entrapment of the cells within the construct, preserving cell viability and morphology.

Additionally, in our system, the 2D condition was the best performer for cell viability, as shown using FACS analysis. Generally, 2D and 3D models are considered equally, relative to this feature [21,22]. However, a significant amount variability among them has been observed, and is mainly related to the stimulus employed (i.e., drug resistance) [22], suggesting that cell viability within the construct strictly depends on cell line perturbation biology, which is not always comparable to the canonical 2D model. It is also likely that Ker-AuNPs might induce a mechanical effect on cell membranes for long-term cultures, slightly reducing the cell viability.

Finally, Ker-AuNPs are also functional in our system, as PTT experiments have confirmed their excellent properties for producing photo-thermal heating. We found that the temperature increased over time, with a peak at 56 h, suggesting the latter as being the optimal timing for preserving cell viability, internalization, integration on the 3D construct, and thermal properties. Notably, the laser-assisted PTT experiments also highlight that 3DB samples cultured for 56 h possess a higher amount of Ker-AuNPs, confirming the immunofluorescence analysis.

## 4. Materials and Methods

### 4.1. Synthesis of Fluorescent Labeled Ker-AuNPs

Spherical AuNPs with a diameter of about 25 nm were synthesized by exploiting the Turkevich method [23], using sodium citrate as a reducing agent. After the synthesis, a solution of AuNPs (1.5 mM) was mixed with a solution of keratin to have a 1:100 weight ratio between Au and keratin. An amount of keratin three orders of magnitude higher than what was expected to be conjugated was used to guarantee that all the AuNP binding sites were saturated. The solution was gently shaken overnight, then the AuNPs were centrifuged twice at 14,300× *g* and resuspended in MilliQ water to remove unbound keratin. Fluorescently labeled Ker-AuNPs were realized by first labeling the keratin solution using the FluoReporter FITC Protein Labeling Kit (ThermoFisher, Whaltam, MA, USA). After preparing a buffer solution of NaHCO_3_ 1 M, pH 9, 1955 µL of buffer and 45 µL of keratin solution, 44 mg/mL, were mixed to obtain 2 mL of solution with a final concentration of keratin of about 1 mg/mL. FITC powder was dissolved in 10 µL of DMSO. The solubilized FITC was added to the buffered keratin solution and mixed using a stirrer for 1 h under dark conditions at room temperature. Subsequently, the keratin–FITC solution was dialyzed in a dialysis cassette with a molecular weight cut-off (MWCO) of 3500 kDa for three days against MilliQ water. Ker*-AuNPs were synthesized using a similar protocol used for Ker-AuNPs.

### 4.2. UV-Vis Spectroscopy

A UV-Vis spectrophotometer (Lambda 365 from PerkinElmer, The software used for data acquisition and analysis was UV WinLab, from PerkinEelmer, Waltham, MA, USA) was used to acquire the absorption spectra of the samples.

### 4.3. Fluorescence Spectroscopy

The fluorescence of the Ker-AuNPs was measured with a Fluoromax ^®^-4 spectrofluorometer (Horiba Jobin-Yvon Inc., Montpellier, France), using an excitation wavelength of 491 nm to measure fluorescence, and an emission wavelength at 515 nm for the excitation spectra.

### 4.4. Fluorescence Microscopy

Fluorescence microscopy images of fluorescent-labeled Ker-AuNPs were collected using a fluorescence microscope ZEISS Axiolab 5 (Images were acquired and processed by the ZEN core software (Carl Zeiss, Göttingen, Germany)) equipped with fluorescence modules. 

### 4.5. Transmission Electron Microscopy (TEM) Analysis

TEM images were acquired using a JEOL JEM-1011 (100 kV) apparatus after drop-casting the samples onto a plasma-cleaned Cu grid coated with ultrathin carbon. The solution was left to dry for 24 h before imaging the samples [24].

### 4.6. U87-MG Cell Culture

Human U87-MG glioblastoma cells were purchased from ATCC and grown as already reported [25]. Briefly, cells were maintained in Dulbecco’s modified Eagle’s medium (DMEM, Gibco) supplemented with 10% heat-inactivated fetal bovine serum (FBS) (Gibco), 100 U/mL penicillin, and 100 mg/mL streptomycin (EuroClone) in a humified atmosphere with 5% CO_2_ at 37 °C. To obtain a sufficient amount of cells for the bioprinting process, the medium was changed every three days, and cells were detached using trypsin–EDTA only when confluent and expanded.

### 4.7. Bioink Formulation

Gelatin (type A3 from porcine skin) Methacryloyl (GelMa) was used as a hydrogel to perform the three-dimensional bioprinting experiments, and was prepared using a reaction of gelatin with methacrylic anhydride [26]. The stock solution of bioink was prepared by dissolving 6% *w/v* low molecular weight Alginate (ALG, FMC Biopolymers) and 6% *w/v* of GelMA in 1 mL of HEPES 25 mM (Sigma-Aldrich). The obtained solution was then filtered (0.22 µm) to ensure sterility. GelMA and ALG, employed for the formulation of the bioink, and play different roles in the 3D bioprinting process. ALG was used only as a temporary material template to allow a precise deposition of the hydrogel fibers loaded with cells through a custom-built co-axial printing head, delivering simultaneously the bioink and a cross-linking solution of CaCl_2_. GelMa, after UV-light exposition, generated a covalently cross-linked matrix in which embedded cells can spread, proliferate, and differentiate. Subsequently, the cells were trypsinized, centrifuged, and resuspended in a freshly prepared bioink stock solution to a final concentration of 12 × 10^6^ cells/mL. Finally, the bioink was added with the fluorescent-labeled Ker-AuNPs at a final concentration of 75 μM and 1 mg/mL of Irgacure 2959 photoinitiator.

### 4.8. 3D Bioprinted Constructs

The preformulated bioink and a calcium chloride solution (0.3 M) were loaded in 1 mL sterile Hamilton glass syringes, and placed on microfluidic pumps [26]. For all experiments, 10 layers-thick constructs were generated, with layers being perpendicular to each other, and a 50 μm distance between the hydrogel stripes in the X–Y plane. The final result was a bioprinted scaffold with a dimension of 8 × 8 × 1 mm^3^. After 3D bioprinting, all scaffolds were collected, placed in a 60 mm dish, and cross-linked with UV light at low light intensity (365 nm, 4–5 mW/cm^2^) for 5 min. Then, the samples were washed with 25 mM HEPES buffer containing 5 mM EDTA for 3 min, to remove as much ALG as possible, and cultured in a growth medium. The constructs were used to perform the experiments at different time points, i.e., 24 and 56 h after the bioprinting process. The 0 h timepoint referred to samples digested immediately after UV cross-linking. Cells in the 2D standard condition were used as a control group.

### 4.9. Viability Assessment

The cells were extracted from the bioprinted constructs and analyzed using flow cytometry. Briefly, the samples were treated with a digestion solution composed of 300 U/mL collagenase II (337 U/mg; Worthington) and 0.65 U/mL collagenase D (0.29 U/mg; Sigma-Aldrich, St. Louis) in HBSS buffer for 10 min at 37 °C. The digestion process was blocked using a stop solution containing 1 × PBS enriched with 5% FBS. Then, the cells were centrifuged at 1200 rpm for 5 min at RT and resuspended in LIVE/DEAD Fixable Aqua Dead Cell Stain Kit-Pacific Orange (1:1000, Invitrogen). After 30 min at RT, the cell suspension was washed with 1 × PBS and acquired using a FACS Canto II cytometer (Becton Dickinson, BD). The data analysis was performed with FlowJo V10.8.1 software (BD Life Sciences; Franklin Lakes, NJ, USA).

### 4.10. Immunofluorescence Assay

The bioprinted constructs used for the immunofluorescence assay were generated using fluorescent U87-MG cells and Ker-AuNPs. Indeed, the cells were labeled with the lipophilic tracer DiI (1: 5000, Invitrogen) for 20 min at 37 °C, and the nuclei were counterstained with Hoechst (1:1000) for 15 min. Then, the U87-MG cells were washed twice with PBS, trypsinized, centrifuged, and encapsulated in the bioink stock solution at 12 × 10^6^ cells/mL. The fluorescent Ker-AuNPs were resuspended in the bioink. After 24 and 56 h of culture, the obtained 3DB constructs were washed with 1 × PBS for 10 min and fixed in 4% PFA for 1 h.

A Leica SP5 laser scanning confocal microscope was used to acquire 3DB samples. Volumetric 3D reconstruction was achieved for better in-depth visualization on 300 μm^3^ volumes. A total of 1–3 areas at 10× (30 steps Z10mm) were acquired. The relative magnifications were obtained with a crop. The images were further processed and elaborated via NIS-Elements v.5.11 software (Leica Microsystem, Wetzlar, Germany).

### 4.11. Statistical Analysis

Statistical analysis was carried out using Prism 8 (GraphPad Software, La Jolla, CA, USA). Data are presented as mean ± Standard Deviation (SD). The statistical significance was assessed using ordinary one-way ANOVA with Tukey correction to evaluate the differences between means. A *p*-value < 0.033 (*), 0.002 (**), and 0.001 (***) was considered statistically significant.

### 4.12. Transmission Electron Microscopy for Ultrastructural Cells Analysis

U87-MG cells incubated with Ker-AuNPs at 75 μM for 24 h were detached and transferred to the Eppendorf tubes for TEM characterization. After the centrifugation process, the cell pellet was fixed with 2.5% glutaraldehyde (SIC, Rome, Italy) in 0.1 M PBS for two days at 4 °C, and then rinsed with PBS solution. Next, the samples were post-fixed using 2% osmium tetroxide (Agar Scientific, Stansted, UK) for 2 h and rinsed again in PBS [27]. The samples were dehydrated via exchange with ethanol, immersed in propylene oxide (BDH Italia, Milan, Italy) for solvent substitution, and embedded in epoxy resin Embed-812 (SIC, Rome, Italy). Using an ultramicrotome (Leica EM UC6, Vienna, Austria), ultrathin (80–90 nm) sections were obtained.

The ultrathin sections were collected on 100-mesh copper grids (Assing, Rome, Italy) stained with a mix of lanthanides solution (Uranyless, Electron Microscopy Sciences) and lead citrate [28]. Imaging was performed using a TEM apparatus (Carl Zeiss EM10, Thornwood, NY, USA) with an accelerating voltage of 60 kV and a DEBEN XR80 AMT CCD camera.

### 4.13. Photo-Thermal Setup

The thermo-optical setup used for the experiments makes use of a CW laser (gem532, Laser Quantum) operating at 532 nm (highest absorption band of Ker-AuNPs) and a high-resolution thermal camera (FLIR, A655sc) equipped with a close-up IR lens (2.9× magnifying factor, working distance, 15 cm). The samples were illuminated at normal incidence from the side, and the thermal images were collected from the same side, with a slight angle to the normal incidence direction.

## 5. Conclusions

We have reported a combination of a 3DB glioblastoma tumor model and biomimetic Ker-AuNPs for PTT applications that mimic more reproducible in vivo-like conditions. To this end, U87-MG cells cultured with fluorescent-labeled Ker-AuNPs have been bioprinted in a 3D architecture. Morphological, optical, and viability assay experiments, and thermo-optical studies have confirmed the capability of U87-MG cells to interact with Ker-AuNPs, highlighting a correlation between the stability of the 3D culture over time, and the thermo-optical response.

One of the main potential for 3DB is represented by the capability to print separately during the same process for both cells and Ker-AuNPs. This exciting possibility enables the localization of Ker-AuNPs in specific areas of the 3D constructs, thus mimicking selective accumulation in specific sites (e.g., tumor sites).

Ongoing and future experiments will be centered to test Ker-AuNPs with more exotic geometries such as gold nanorods (AuNRs) [29] and bipyramids (AuBPs) [30]. Both AuNRs and AuBPs exhibit higher photo-thermal efficiency and absorption in the first biological window (700–900 nm), where tissue absorption is low, showing higher light penetration (1–2 cm). In addition, ongoing studies are devoted to developing innovative biomimetic NPs made with different chemical compositions, such as Ag [5] and CuS [31,32]. This crucial aspect enables the overcoming of several critical issues [33] and the tackling of new nanomedicine challenges, such as exploiting the synergistic effects of chemotherapeutic agents and PTT. Upcoming research is also devoted to fully exploiting the potential of 3DB by combining cancer and healthy cells in complex architectures to investigate whether Ker-AuNPs have the inherent capability to accumulate selectively in cancer cells without producing PTT effects in healthy cells.

## Supplementary Materials

The following supporting information can be downloaded at: https://www.mdpi.com/article/10.3390/ijms23179528/s1.
